# New miRNA labeling method for bead-based quantification

**DOI:** 10.1186/1471-2199-11-44

**Published:** 2010-06-16

**Authors:** Alberto Biscontin, Silvia Casara, Stefano Cagnin, Lucia Tombolan, Angelo Rosolen, Gerolamo Lanfranchi, Cristiano De Pittà

**Affiliations:** 1 Department of Biology and CRIBI Biotechnology Centre, Università degli Studi di Padova, Via U. Bassi, 58/B, 35121 Padova, Italy; 2 Clinica di Oncoematologia Pediatrica, Azienda Ospedaliera-Università degli Studi di Padova, Vi Giustiniani 3, 35128 Padova, Italy

## Abstract

**Background:**

microRNAs (miRNAs) are small single-stranded non-coding RNAs that act as crucial regulators of gene expression. Different methods have been developed for miRNA expression profiling in order to better understand gene regulation in normal and pathological conditions. miRNAs expression values obtained from large scale methodologies such as microarrays still need a validation step with alternative technologies.

**Results:**

Here we have applied with an innovative approach, the Luminex^® ^xMAP™ technology validate expression data of differentially expressed miRNAs obtained from high throughput arrays. We have developed a novel labeling system of small RNA molecules (below 200 nt), optimizing the sensitive cloning method for miRNAs, termed miRNA amplification profiling (mRAP). The Luminex expression patterns of three miRNAs (miR-23a, miR-27a and miR-199a) in seven different cell lines have been validated by TaqMan miRNA assay. In all cases, bead-based meas were confirmed by the data obtained by TaqMan and microarray technologies.

**Conclusions:**

We demonstrate that the measure of individual miRNA by the bead-based method is feasible, high speed, sensitive and low cost. The Luminex^® ^xMAP™ technology also provides flexibility, since the central reaction can be scaled up with additional miRNA capturing beads, allowing validation of many differentially expressed miRNAs obtained from microarrays in a single experiment. We propose this technology as an alternative method to qRT-PCR for validating miRNAs expression data obtained with high-throughput technologies.

## Background

MicroRNAs (miRNAs) are endogenous 18-24 nucleotides (nt) long noncoding RNAs (ncRNA) that control gene expression by targeting mRNAs and triggering either translation repression or degradation. The degree of complementarity between a miRNA and its mRNA target determines, at least in part, the regulatory mechanism [1]. Recently, a third less understood mechanism of small RNAs interference on gene expression involves heterochromatin silencing [2]. Many miRNAs are highly conserved among animals and plants [3] and it is estimated that up to 33% of all mRNA coding genes are negatively regulated by miRNAs [4,5]. miRNAs exhibit temporally and spatially regulated expression patterns during diverse developmental and physiological processes and clearly animals cannot survive without miRNAs [6,7]. Most of the miRNAs that have been characterized so far in animals seem to regulate developmental processes, including larval stage transitions and neuronal development in *C. elegans *[8], growth control and apoptosis in *Drosophila melanogaster *[9] or haematopoietic differentiation in mammals. Many miRNAs have been found to display unique tissue [10], developmental stage [11] or disease-specific patterns [12]. These observations imply that each tissue is characterized by a specific set of miRNAs that contribute to the definition of the features of that tissue. Hundreds of novel conserved and non-conserved microRNAs have been identified by bioinformatic analyses, suggesting that the total number of human miRNAs could reach 1,000 [13]. According to recent computational predictions, as many as 200 mRNAs can be regulated by a single miRNA, which implies that over one third of protein-coding genes in humans are regulated by miRNA [14,15].

So, the ability to monitor changes in miRNAs expression is important for understanding gene regulation both in physiological and pathological conditions. Several methodologies have been adapted for profiling miRNA expression: northern blotting with radiolabeled probes [16,17], cloning [18,19], massive parallel signature sequencing (MPSS) [20], quantitative PCR-based amplification of precursor [21] or mature miRNAs [22], SAGE-based techniques [23], bead-based profiling methods [24,25] and oligonucleotide microarray [26]. Microarray technology has been successfully used for evaluating variations of miRNA expression during development [27,28], differentiation [29], oncogenesis [30-32], disease progression [33,34] and for the primary identification of new miRNAs that were predicted by bioinformatic approaches [35,36]. However, at present, no standard methodology exists for hybridization-based profiling of miRNAs and, as a consequence, comparison of expression data from different experiments can be difficult. To solve these problems it will be necessary to develop quality procedures for miRNA microarrays. Furthermore, miRNA expression obtained from high throughput arrays has to be validated with alternative technologies. Quantitative real-time PCR (qRT-PCR) has become the golden standard of miRNA quantification because it offers the highest sensitivity from small amounts of starting material and it is able to detect as less as 1-nt difference between miRNAs. Another interesting method is the Luminex^® ^xMAP™ system that is a multiplexed microsphere-based suspension array platform capable of performing and reporting up to 100 different analyses in a single reaction vessel [37,38]. In particular, oligonucleotide-capturing probes complementary to miRNAs of interest are coupled to carboxilated 5-micron polystirene beads impregnated with variable mixtures of two fluorescent dyes, each representing a single miRNA. Using this technique Lu and colleagues [24] were able to differentiate tumours that were instead inaccurately classified by mRNA profiles. Recently, a study performed with Luminex miRNA platform, identified new markers of human breast cancer subtype [39]. When compared to glass-slide microarrays, the bead-based miRNA arrays show many advantages such as easy of use, low cost, superior statistical performance, faster hybridization kinetics (solution hybridization) and higher flexibility in array preparation. Furthermore, the Luminex bead array system has been used in a wide range of multianalyte applications throughout the drug discovery and diagnostics fields and it is also widely adopted for quantitative multiplexed protein expression analysis [40,41].

In this study we have applied the Luminex^® ^xMAP™ technology in a novel approach to validate expression data of differentially expressed miRNAs obtained from high throughput arrays. We have developed an innovative system for labeling of small RNA molecules (below 200 nt) optimizing the sensitive cloning method for miRNAs, termed mRAP, developed by Takada and Mano to define mouse miRNA transcriptional signature [19,42,43]. The expression patterns of three miRNAs (miR-23a, miR-27a and miR-199a) measured by our Luminex approach in seven different cell lines were validated by TaqMan miRNA assay. In all cases, the two technologies gave super imposable results. Our data demonstrate that bead-based detection of individual miRNA is a feasible approach, associated to high speed and low cost. The Luminex^® ^xMAP™ technology is also feasible for multiplexing, since several beads prepared to capture different miRNAs can be added in the same reaction allowing the validation of many differentially expressed miRNAs obtained from large-scale approaches in a single experiment.

## Results and Discussion

### miRNA expression in rhabdomyosarcoma cell lines

Rhabdomyosarcomas (RMS) are rare but very aggressive tumours of childhood that arise as a consequence of regulatory disruption of the growth and differentiation pathways of myogenic precursor cells [44]. Based on morphology, two major RMS subtypes can be identified: embryonal RMS (ERMS) and alveolar (ARMS). To better understand the global function of miRNA in RMS, we analyzed the expression profile of 7 different RMS cell lines (3 ARMS and 4 ERMS) using the *mirVana *miRNA Probe Set V1 (Ambion) that is a collection of about 400 amino-modified DNA oligonucleotides [45]. Briefly, the miRNA population from a single cell line was compared to a reference sample consisting of a pool of the seven small RNA samples ( < 200 nt) mixed in equal amounts. The miRNA microarray platform was able to distinguish *PAX3-FKHR *positive (RH4, RH30) and negative RMS (RD, CCA, SMS-CTR, RH36, RH18) cell lines through the expression pattern of about 120 miRNAs (data not shown). Since the translocation positive RMS patients fared worse than the negative counterpart [46] our results demonstrated the potential of miRNA expression profiling to classify different RMS subtypes, in agreement to previous gene expression studies [47-49], and set the basis for a further functional characterization of selected miRNAs implicated in RMS pathogenesis and in the different clinical behaviour and aggressiveness of the two RMS subtypes. We decided to study miRNAs with the greatest difference in expression between *PAX3-FKHR *positive and negative RMS. So expression levels for three discriminant miRNAs (miR-23a, miR-27a and miR-199a) were validated by xMAP™ technology and TaqMan qRT-PCR.

### Testing the hybridization specificity and sensitivity between targets and capture probes coupled to microspheres

**Figure 1:**
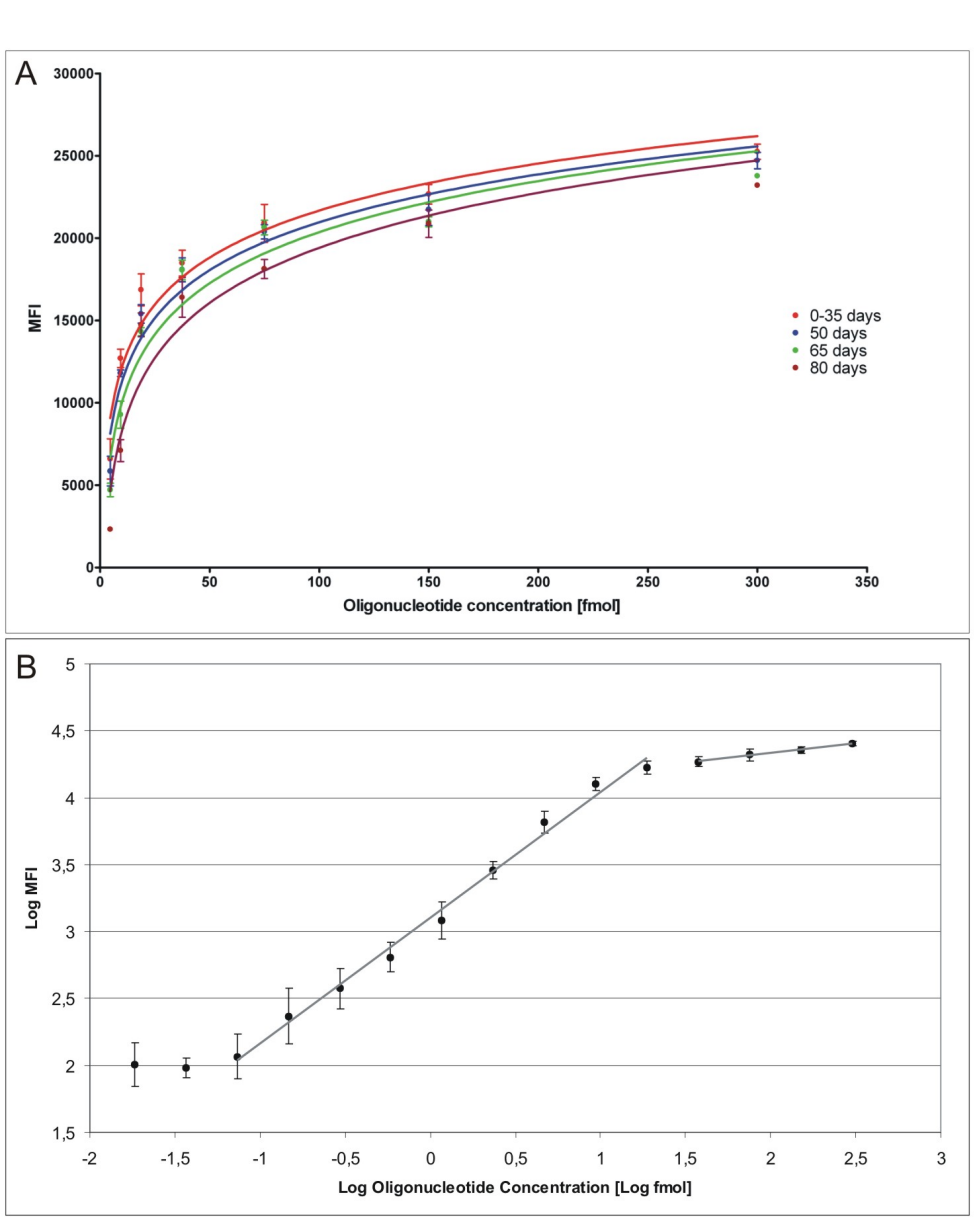
**Stability of conjugated microspheres and sensitivity of hybridization**. **A**) Increasing amounts of miR-199a target oligonucleotide (from 15 amol to 300 fmol) show higher fluorescence levels reaching a plateau when the target is present at high concentrations. The curve shows a logarithmic trend. We observed a slightly decrease of the hybridization signal at 50, 65 and 80 days after conjugation. **B**) Calibration curve (0-35 days) of miR-199a in a log-scale from 15 amol to 300 fmol. The two lines show the linear and plateau range of fluorescence levels representing by two different slopes: 0.94 and 0.15 respectively with R^2 ^= 0.99. The linearity range of our technique is comprised between a lower value of 0.073 fmol and an upper value of 18.75 fmol. Mean value of expression and 95% confidence intervals are associated to each fluorescence value (MFI corresponds to Median Fluorescence Intensity).

**Figure 2:**
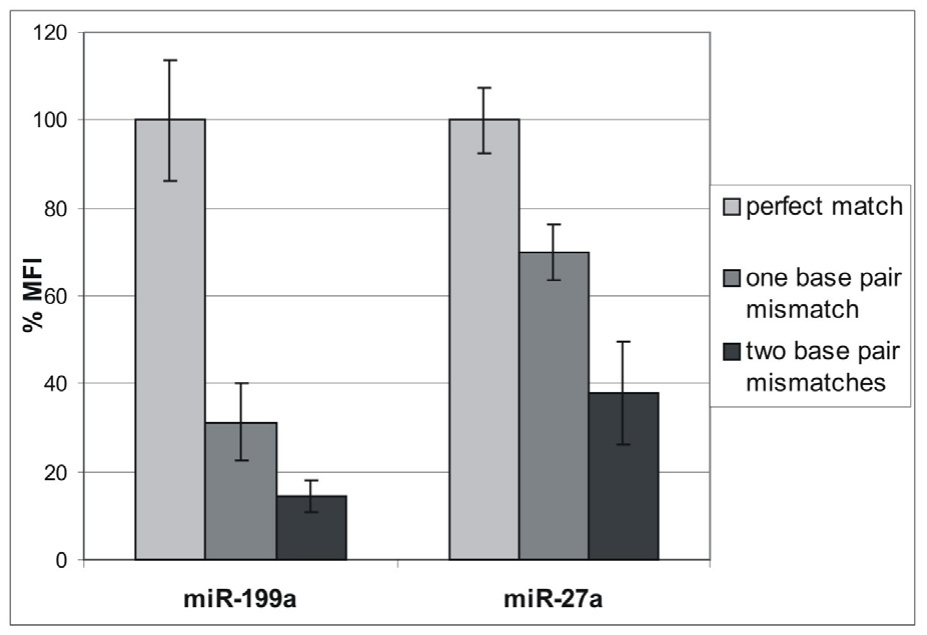
**Specificity of hybridization between capture probes and miRNA targets**. Biotynilated miR-199a and miR27a (bottom panel) were captured in solution by the corresponding probes with exactly matching sequences (light grey boxes) or with one (dark grey boxes) or two mismatched bases (black boxes). The fluorescence signal obtained by the hybridization of both the perfectly matching probes was set to 100%. The hybridization signal between both capture probes and the corresponding miRNA target shows a statistically significant decrease (p-value < 0.05) when one or two mismatches are introduced into the capture sequence. Hybridization experiments were obtained with 18.75 fmol of biotynilated targets that fall in the linearity range of our method as shown in Figure 1B. Mean value of expression and 95% confidence intervals are associated to each fluorescence value (%MFI corresponds to the percentage of Median Fluorescence Intensity).

To prepare the capture probes, 21-23 bases-long oligonucleotides with sequence complementary to each of the three differentially expressed miRNAs (miR-23a, miR-27a and miR-199a) in RMS cell lines, were synthesized and coupled to different color-coded microspheres in separate reaction tubes and then mixed for multiplexed assays. The targets were oligonucleotides complementary to capture probes and tagged with biotin at their 5'-end. Capture probes (approximately 5,000 beads for each probe) were mixed in the same tube with targets at various amounts ranging from 15 amol to 300 fmol. Phycoerythrin (PE)-coniugated streptavidin was added to the reaction mixture to detect bound targets that were biotynilated. The signal of each target hybridized to its specific capture probe coupled to microspheres was determined by the fluorescence intensity of phycoerytrin. At least 100 microspheres of each set were analyzed by the Bio-Plex™ system to obtain a median fluorescence intensity value (MFI) that was representative of the whole population of each set of beads. As shown in Figure 1A, the hybridization signal for miR-199a with capture probe varied with the amounts of added target in a logarithmic trend, reaching a plateau when the targets were present at the highest concentrations. Furthermore, the specificity of hybridization did not change while increasing from 1 to 3 the number of different microspheres in the reaction. We have evaluated the stability of conjugated microspheres at regular intervals during 80 days after conjugation. The hybridization signal is stable up to 35 days. As shown in Figure 1A, the hybridization signal for the miR-199a slightly decreased with the distance from conjugation, but the hybridization between the capture probe and the corresponding target remained specific and proportional. Furthermore, our data show that it is better to use microspheres conjugated in the same day to correctly detect miRNA expression values. We have also represented the curve in a log-scale (Figure 1B) highlighting the linearity range of our technique: the lower limit of sensitivity is 0.073 fmol and the upper is 18.75 fmol. We have obtained comparable results with either miR-23a or miR-27a. We measured the specificity of our technique by testing the variation in hybridization signal intensity when miR-27a and miR-199a are captured with probes that contain one or two mismatches in their complementary sequences (see Table 1). We have evaluated the signal of each target (biotynilated oligonucleotides perfectly complementary to mature miRNA sequence) hybridized to the correspondent capture probes with perfect match, one or two mismatches. As described above, capture probes were mixed in the same tube with targets at various amounts (from 15 amol to 300 fmol). Figure 2 shows that for both miRNAs we obtain a significant decrease of hybridization fluorescence intensity with capture probes containing a single mismatch. The signal drop is even greater with two mismatches: 62% for miR-27a and 85% for miR-199a.

**Table 1:** Capture probes

ID	Nucleotide sequence (5'-3')	Lenght (nt)
**miR-23a**	GGAAATCCCTGGCAATGTGAT	21
**miR-27a**	GCGGAACTTAGCCACTGTGAA	21
**miR-27a-1MM**	GCGGAACT**A**AGCCACTGTGAA	21
**miR-27a-2MM**	GCGGAA**G**TTAGCCACTG**A**GAA	21
**miR-199a**	GAACAGGTAGTCTGAACACTGGG	23
**miR-199a-1MM**	GAACAGGTAGTCTGAA**G**ACTGGG	23
**miR-199a-2MM**	GA**T**CAGGTAGT**G**TGAACACTGGG	23
**Spike 18**	CATTGCCACAATCAAGACTAAGA	23

Nucleotide sequence of capture probes synthesized with 5'-amino linker and a C12 spacer. Basepair mismatches (named MM) are in bold and underlined

**Figure 3:**
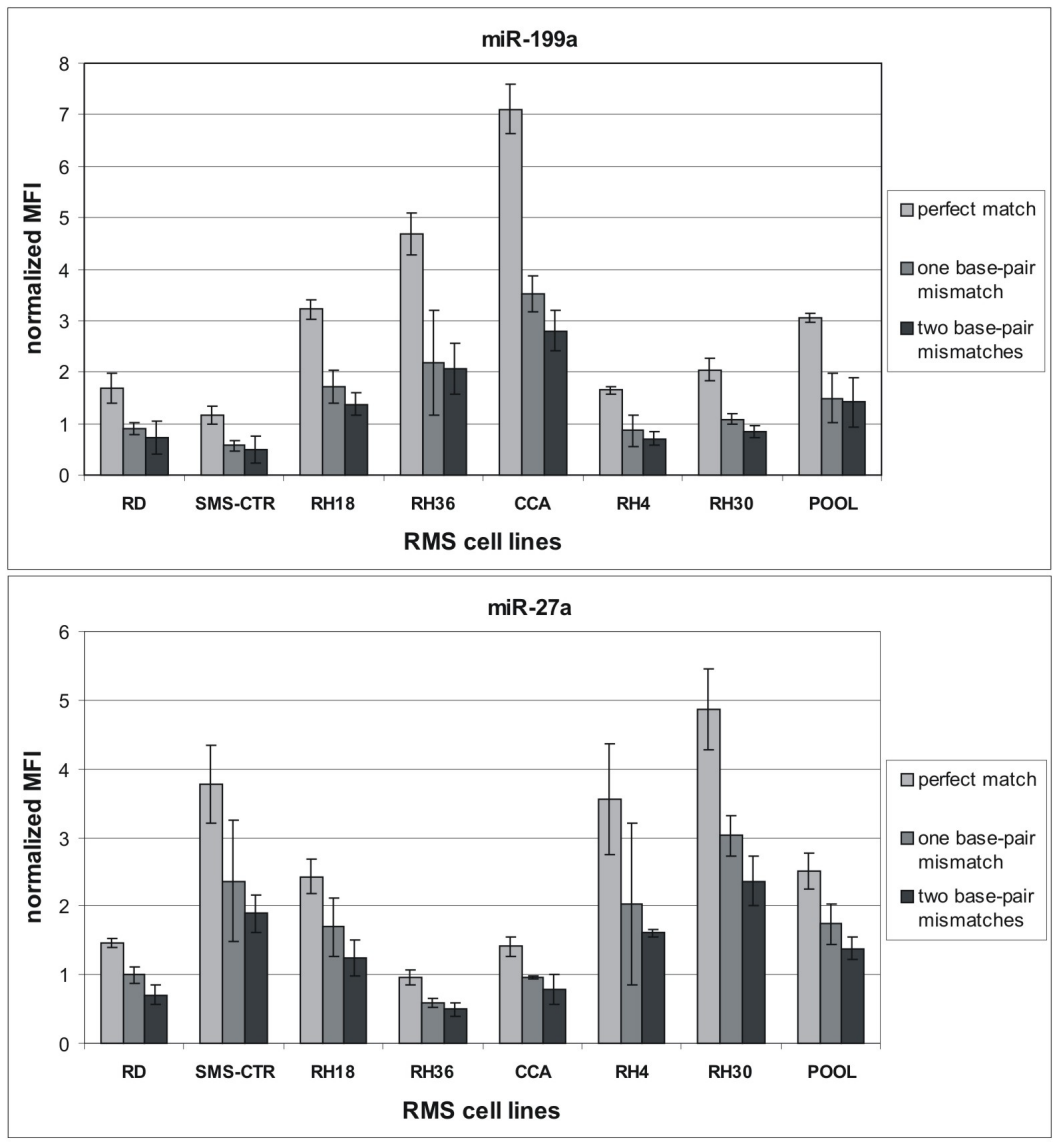
**Specificity of hybridization between capture probes and miRNA targets in complex RNA populations**. miR-199a (top panel) and miR27a (bottom panel) were captured from labeled total RNA populations prepared from 7 different RMS cell lines (RD, SMS-CTR, RH18, RH36, CCA, RH4, RH30) and a pool these RNA preparations mixed in equal amounts (POOL) with capture probes with exactly matching sequences (light grey boxes) or with one (dark grey boxes) or two mismatched bases (black boxes). The hybridization signal in all complex RNA mixtures between both capture probes and the corresponding miRNA target in comparison to the reference pooled sample shows a statistically significant decrease (p-value < 0.05) when one or two mismatches are introduced into the capture sequence. Mean value of expression and 95% confidence intervals are associated to each fluorescence value (normalized MFI represents the average of the ratio between the value of fluorescence of miRNAs and the spike).

We have also determined the expression levels of above cited miRNAs, using the capture probes with perfect match, one or two mismatches in complex RNA populations obtained from seven RMS cell lines (RD, CCA, SMS-CTR, RH36, RH18, RH4, RH30) in comparison to a reference sample (Pool). As shown in Figure 3, we observe a decreasing trend in fluorescence signal for the capture probes with two mismatches. These experiments show that the specificity of a capture probe is proportional to the number of mismatches present in its sequence and that this specificity guarantee the correct quantification of miRNA expression levels in complex RNA populations.

### A new application of mRAP method for miRNA labeling

**Figure 4:**
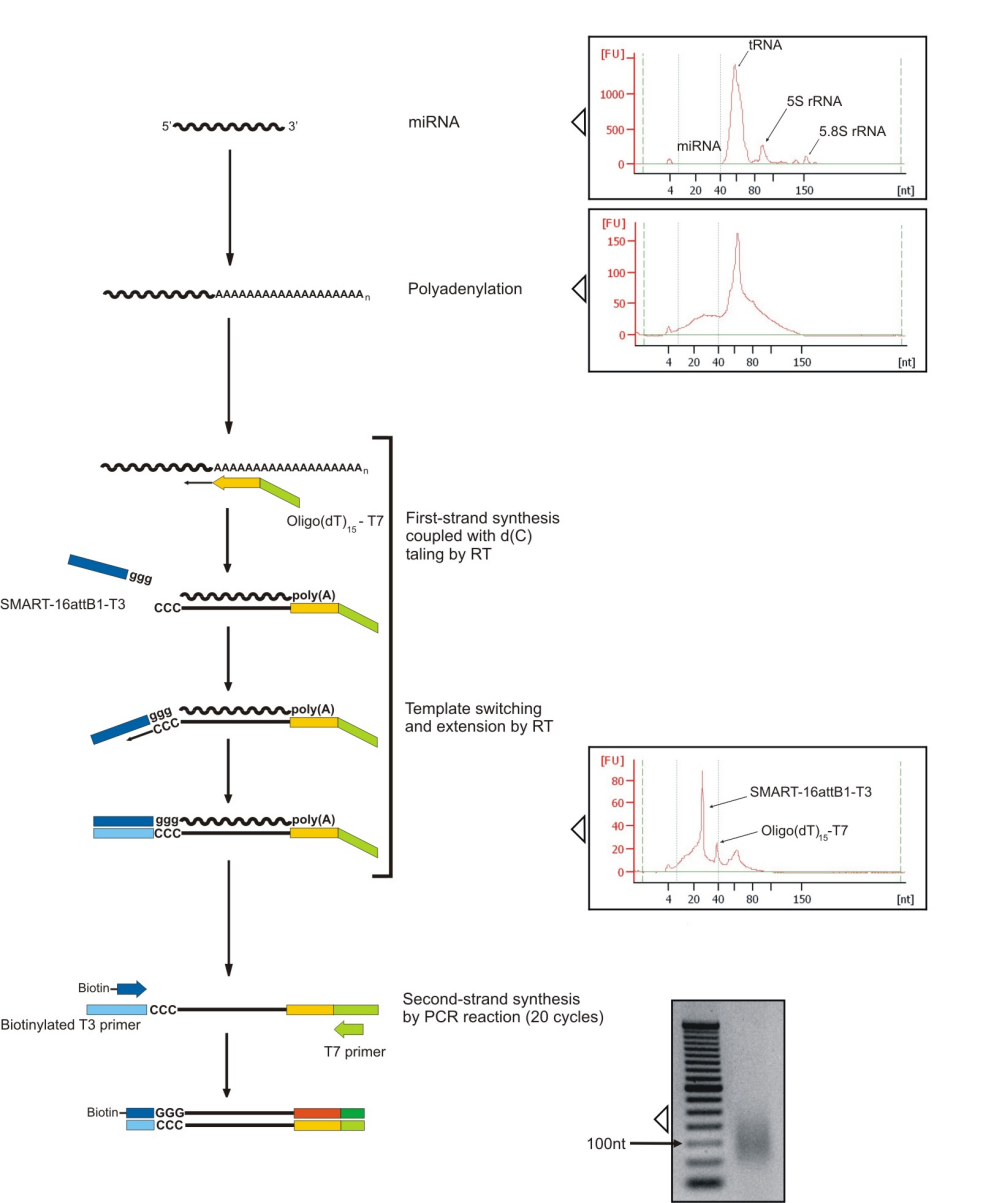
**Schematic representation of miRNA labeling method for bead based quantification**. All protocol steps and quality checking with Agilent Bioanalyzer 2100 and gel electrophoresis (2% agarose) are schematically represented: starting material (RNA < 200 nt), the result of polyadenylation reaction with PAP, the synthesis of first-strand cDNA (peaks are associated to Oligo-dT15-T7 and SMART-16attB1-T3 primers) and the second-strand synthesis by PCR amplification.

We have developed a new sensitive miRNA labeling method based on the mRAP strategy recently developed by Mano and Takada for miRNA cloning [40,41]. This new procedure is described schematically in Figure 4. Small RNA molecules ( < 200-nt) purified by PureLink™ miRNA Isolation Kit (Invitrogen) were polyadenylated with Poly(A) polymerase (PAP). Complementary DNAs corresponding to the miRNAs were then synthesized with the use of reverse transcriptase and a RT primer, named Oligo-dT_15_-T7, complementary to the poly(A) sequences added to the miRNA. This RT reaction was allowed for 30 minutes in order to synthesize cDNA molecules of about 200 nt. We have used a degenerated oligo(dT) to reduce the length of the neo-synthesized poly(A) tails to 15 nucleotides. Given that some reverse transcriptases possess terminal deoxynucleotidyl transferase activity, the synthesized cDNA strands frequently result with small poly(C) overhangs at their 3' ends. After annealing a long 5'-adptor, named SMART-16attB1-T3, to such poly(C) overhangs, PCR was used to amplify the miRNA-derived cDNAs. Using a T3-biotinylated forward primer, we have obtained biotinylated cDNA that was detected with Phycoerythrin (PE)-coniugated streptavidin after hybridization reaction. Every step of this protocol was quality checked by Agilent Bioanalyzer 2100 and the resulting electropherograms are represented in Figure 4. Electropherogram analysis of the RT products have revealed two major bands of ~30 and ~40 nt that correspond to the 5'-adaptor (SMART-16attB1-T3) and the oligo-dT_15_-T7 primer respectively. The electropherograms also evidence the action of degenerated oligo(dT) that reduces the length of neo-synthesized cDNAs to 100-110 nucleotides by shortening the poly(A) to 15 nt (Figure 4).

**Figure 5:**
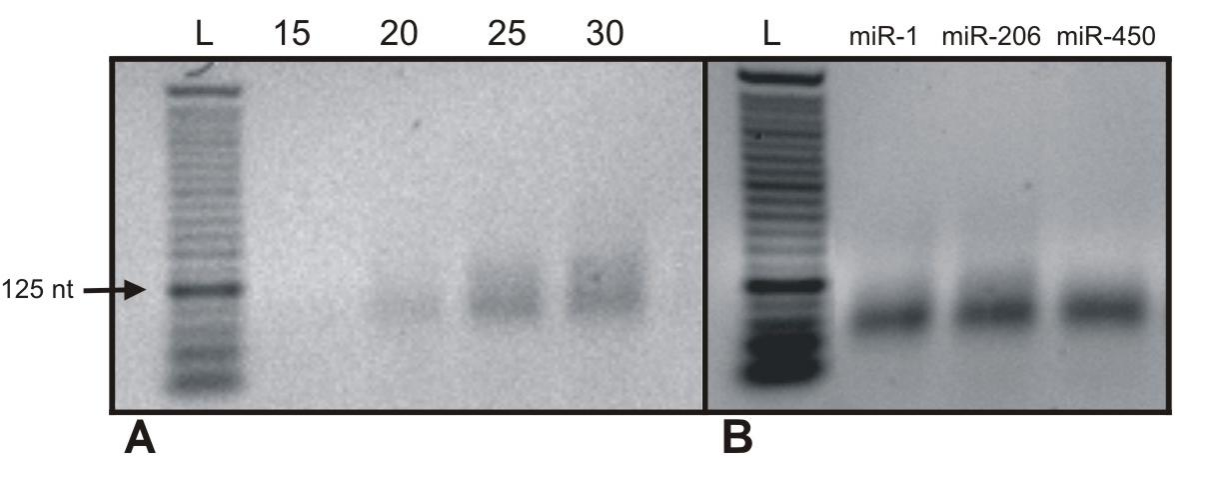
**Second-strand synthesis by PCR reaction**. **A**)Determination of the optimal PCR amplification cycle: the 2% agarose gel shows the amplification products of RH30 first-strand cDNA stopped at 15, 20, 25 and 30 PCR cycles. **B**) Presence of specific miRNAs in the labeled cDNA population: the 2.25% agarose gel shows PCR amplification products of three specific miRNAs (miR-1, miR-206 and miR-450) performed by using the sequence of each miRNA as forward primer, and the sequence for T7 promoter, which is common to all labeled molecules, as reverse primer.

We have decided to block PCR reaction during the exponential phase at 22 cycles, to avoid distortion of the actual concentration of miRNAs in the sample under consideration. We have demonstrated that PCR cycles between 20 and 25 are sufficient to achieve a good level of amplification without reaching the plateau phase in which the expression differences would be invalidated (Figure 5A).

Finally, to verify the presence of miRNAs within the labeled cDNA population we have identified, by PCR, three specific miRNAs (miR-1, miR-206 and miR-450) using the sequence of each miRNA as forward primer, and the sequence for T7 promoter, which is common to all labeled molecules, as reverse primer. The result of PCR amplification of the three miRNAs is shown in Figure 5B.

### Validation of differentially expressed miRNAs by Luminex^® ^xMAP™ technology

Using the xMAP™ assay described above we have determined the expression levels of miR-23a, miR-27a and miR-199a in seven RMS cell lines (RD, CCA, SMS-CTR, RH36, RH18, RH4, RH30) in comparison to a reference sample consisting of a pool of small RNA from each cell line mixed in equal amounts.

**Figure 6:**
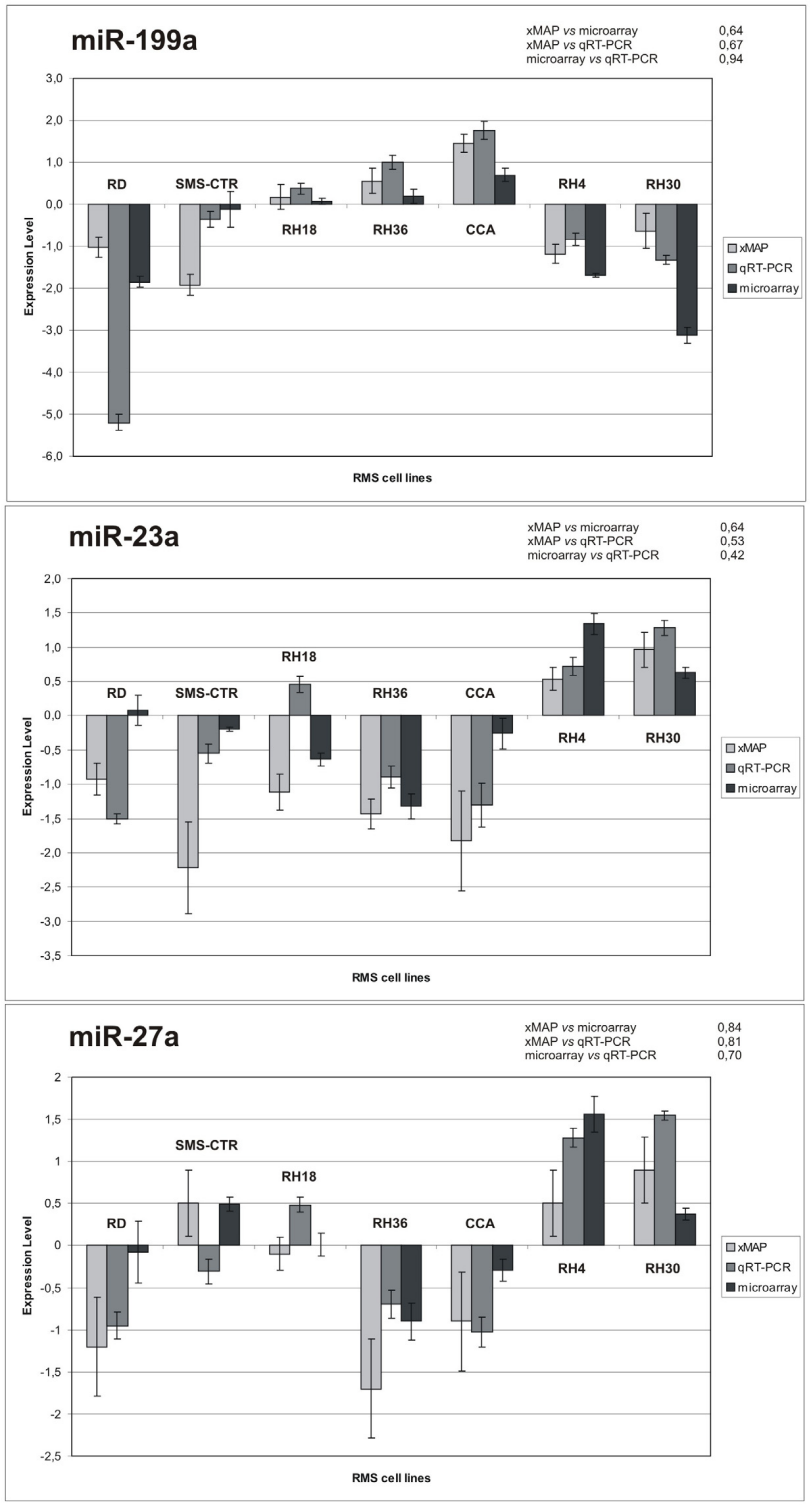
**Comparison of three different techniques used to define miRNA expression profiles in seven RMS cell lines**. Expression levels of miR-199a, miR-23a and miR-27a in seven RMS cell lines obtained with three methods (xMAP™, qRT-PCR and microarray) are represented. Mean value of expression and 95% confidence intervals are associated to each miRNA. The Figure also indicate, for each tested miRNAs, non-parametric Spearman correlations for the following paired comparisons: xMAP™ *vs*. microarray, xMAP™ *vs*. qRT-PCR and qRT-PCR *vs*. microarray.

To evaluate the validity and feasibility of an assay, it is necessary to compare data with that obtained by other established technologies such as, in this case, microarray and qRT-PCR. As shown in Figure 6, the trends of expression levels measured for all the miRNAs by the three technologies (microarray, xMAP™ and qRT-PCR) were very similar. Although the actual values of the relative miRNA hybridization signals in the seven RMS cell lines were not exactly the same for the three technologies, up-regulated miRNAs (miR-23a and miR-27a) in *PAX3-FKHR *positive RMS samples (RH4, RH30) detected originally by microarray were also up-regulated as detected by xMAP™ and qRT-PCR, and signals for miRNAs down-regulated in *PAX3-FKHR *negative RMS samples (RD, CCA, SMS-CTR, RH36, RH18) resulted consistently low by all three independent technologies. To better understand which are the techniques that provide the most similar results we have applied non-parametric Spearman correlation to the following paired comparisons for each tested miRNAs: xMAP_TM _*vs*. microarray, xMAP_TM _*vs*. qRT-PCR and qRT-PCR *vs*. microarray. It is interesting to note that data obtained with xMAP™ technology seem to be more similar to those defined by microarray (Spearman correlation: miR-199a = 0.64, miR-23a = 0.64, miR-27a = 0.84) respect to qRT-PCR (Spearman correlation: miR-199a = 0.67, miR-23a = 0.53, miR-27a = 0.81). From this comparison we could observe that the PCR amplification step introduced in xMAP™ labeling method does not affect miRNA expression levels whereas miRNA expression data obtained from < 200-nt RNA molecules (microarray and xMAP™) differ slightly from those obtained from total RNA (qRT-PCR). This is particularly evident by non-parametric Spearman correlation for qRT-PCR *vs*. microarray (miR-199a = 0.94, miR-23a = 0.42, miR-27a = 0.70) with the exception of miR-199a.

## Conclusions

We have described a new method that makes use of xMAP™ technology for the quantitative determination of single miRNAs. This approach quantifies miRNA expression levels based on hybridization of small RNA populations to the probes designed for miRNAs of interest that are coupled to different color-coded microspheres. A flow-cytometer is used to simultaneously measure the hybridization signal associated with the surface of the microspheres and to categorize the color-coded microspheres. The method allows to obtain up to 100 independent measures. This xMAP™ method offers affordable cost, speed, and high flexibility with capability for multiplexed assays that are increasingly needed. It is valuable for applications such as diagnostic detection of disease miRNAs from clinical samples and validation of differentially expressed miRNAs obtained from microarray analysis.

We have evaluated the stability of conjugated microspheres demonstrating that they are stable for about one month. We have also tested the specificity of hybridization between targets and capture probes showing that the introduction of a single mismatch in the sequence of capture probes significantly hampers its hybridization capacity (Figures 2 and 3). These data have demonstrated the specificity of our capture probes to correctly identify and quantify the expression levels of miRNAs. We have developed a new sensitive small RNAs labeling protocol introducing some changes on mRAP strategy for miRNA cloning. This protocol has been applied successfully to validate the expression levels of three miRNAs that were found differentially expressed in positive and negative *PAX3/FKHR *alveolar rhabomyosarcomas by microarray experiments. To demonstrate the reliability of this method, the transcriptional levels of these miRNAs were also determined by qRT-PCR with TaqMan probes. The most significant information which emerges comparing the data obtained by the three different technologies (microarray, xMAP™ and qRT-PCR) is represented by the general overlapping of the expression profiles obtained. It is interesting to note that small differences in miRNAs expression data could depend on the approaches used for total RNA purification rather than on different labeling protocols. In fact, microarray and xMAP™ data obtained from labeling < 200-nt RNA molecules, are more similar compared to qRT-PCR data obtained from labeling total RNA, as demonstrated by the Spearman non parametric correlation.

We think that the xMAP™ technology associated with our new labeling protocol could become an alternative and equally reliable method in the study of expression of a limited number of miRNAs respect to qRT-PCR with TaqMan probes. The comparison of the two experimental approaches (see Table 2) shows that the xMAP™ technique is less expensive and more flexible allowing the simultaneous analysis of a larger number of miRNAs from the same sample. In addition, xMAP™ does not need for specific reverse transcription reactions for each miRNA and provides at least 100 independent measures for each miRNA, improving the statistical power. Moreover, xMAP™ expression data can be normalized respect to a spike fluorescence signal added in known quantities in the early stages of the labeling reaction, allowing the control over all stages of the reaction. In this way there is no need of an endogenous control, like in qRT-PCR, whose representativeness is sample-dependent.

**Table 2:** Comparison of xMAP™ and qRT-PCR techniques

	xMAP™	qRT-PCR
**< 200 nt RNA molecules enrichment**	YES	NO
**RT step**	YES	YES
**Amplification**	YES	YES
**Endogenous control**	NO	YES
**Control spike**	YES	NO
**Multiplexing**	YES	NO
**Liquid-phase hybridization**	YES	NO
**No. of measures**	100	1
**Custom-tailored assay**	YES	NO
**Cost**	Low	High

Probably, a negative aspect of xMAP™ technology is represented by the use of enriched total RNA ( < 200 nt) that requires higher amounts of starting material respect to qRT-PCR. To avoid the enrichment step of < 200 nt RNA molecules, we propose the use of LNA capture-probe oligonucleotides that increase the affinity of the oligonucleotide for its complementary RNA target leading to a significant enhance in stability and specificity of the duplex.

We think that the technology we have developed could be an alternative method to qRT-PCR for validating miRNAs expression data obtained with a large scale technology such as microarray and it could have a wide application in clinical, pharmaceutical, agricultural and environmental studies.

## Methods

### Cell culture

Human alveolar RMS (ARMS) cell lines, positive for *PAX3-FKHR *translocation (RH4, RH30), negative for *PAX3-FKHR *translocation (RH18) and human embryonal RMS (ERMS) cells (RD, RH36, CCA, SMS-CTR) were maintained in modified Eagle's medium (DMEM) containing 10% fetal calf serum, penicillin (100 U/ml), and streptomycin (100 μg/ml) (Invitrogen) at 37°C, 5% CO2 in a humidified incubator.

The human RMS cell lines RH30 and RD were purchased from ATCC (Manassas, VA); RH4 and RH18 were a gift of Dr P.J. Houghton (St Jude Children's Hospital, Memphis, TN); SMS-CTR, RH36, CCA were obtained from Dr M. Tsokos (NCI, Bethesda, MD).

### Isolation of small RNA molecules

Total RNA was prepared from seven cell lines of human rhabdomyosarcoma using a modified TRIzol (Life Technologies Corporation, Carlsbad, CA, USA) protocol for small RNA enrichment. A pellet of about 6 × 10^6 ^- 9 × 10^6 ^cells was dissolved in 1 ml of TRIzol and the supernatant, containing total RNA, was purified by PureLink™ miRNA Isolation Kit (Life Technologies Corporation) that was specifically designed to enrich total RNA preparation for < 200-nt RNA molecules. RNA quantity and quality were assessed by Nanodrop (NanoDrop Technologies, Wilmington, DE, USA) spectrophotometry and microelectrophoresis using Small RNA Nano LabChip by Agilent 2100 bioanalyzer (Agilent Technologies, Palo Alto, CA, USA) respectively.

### miRNA expression profiling and statistical analysis of data

microRNA expression profiling was carried out using the "mirVana Probe Set V1" (Ambion) that is a collection of about 400 amine-modified DNA oligonucleotides representing a panel of the human, mouse and rat microRNAome in the miRNA Registry (miRBase - Release 9). The probes are 42-46 nucleotides (nt) long, with 18-24 nt segment targeting a specific miRNA, and the remaining sequence serving as spacer. We analyzed the expression profiles of 7 different rhabdomyosarcoma cell lines: 3 ARMS (RH4, RH30, RH18) and 4 ERMS (RD, RH36, CCA, SMS-CTR). The miRNA population from each cell line was compared to a reference sample consisting of a pool of the 7 total RNA samples mixed in equal amounts. Two replicates of each experiment were performed using different microarray slides, in which sample and reference RNAs, labeled either with Cy3 or Cy5 fluorochromes, were crossed in both combinations (dye-swapping procedure). miRNAs were labeled with the mirVana Labeling Kit (Ambion) and amine-reactive dyes (GE Healthcare) as recommended by the manufacturer's protocol [45]. Normalization of expression levels of all spot replicates was performed by MIDAW [50]. Principal component analysis, cluster analysis and profile similarity searching were performed with tMev software [51]. One and two class Significance Analysis of Microarray (SAM) allowed to identify differentially expressed miRNAs [52].

### Capture probe and its coupling to microspheres

A sequence of 21-23 nt complementary for each tested miRNAs (listed in the Table 1) was chosen as capture probe and synthesized with 5'-amino linker and a C12 spacer (PRIMM, Milan, Italy). Capture-probe oligonucleotides were covalently linked to carboxylated fluorochrome microspheres (Bio-Rad Laboratories, Hercules, CA, USA) in water-soluble carbodiimide. Specifically, 1 × 10^6 ^carboxylated microspheres were pelleted in a microcentrifuge for 5 minutes at 12,000×g and then supernatant was carefully removed. The dry microspheres were dissolved in 20 μl of a buffer containing 0.1 M MES (Sigma-Aldrich, St. Louis, MO, USA) at pH 4.5. The amino-substituted capture probe was dissolved in molecular biology grade water at a concentration of 100 μM and 0.5 μl of the solution (containing 0.05 nmole of capture probe oligonucleotides) was added to the beads for the coupling reaction. The coupling reaction was performed by adding 2.5 μl of a freshly made solution of 10 mg/ml 1-ethyl-3-(3-dimethylaminopropyl) carbodiimide hydrochloride (EDC) (Pierce, Thermo Scientific, Wilmington, DE, USA) in molecular biology grade water. The mixture of microspheres, capture probes, and EDC was vortexed briefly and incubated at room temperature for 30 minutes in the dark. Occasionally, the reaction was mixed by finger flicking the tube to keep the microspheres in suspension. A second incubation steps was done adding a freshly-made solution of 10 mg/mL EDC in molecular biology grade water. After the coupling reaction, 500 μl of 0.02% Tween 20 (Sigma-Aldrich) was added to the microspheres. The solution was mixed well by vortex and centrifuged for 6 minutes at 12,000×g. The supernatant containing free-capture-probe oligonucleotides and excess EDC was carefully removed. The coupled microspheres were washed in 500 μl of 0.1% SDS (Sigma-Aldrich) by vortex and centrifuged for 5 minutes at 12,000×g. Finally, the supernatant was removed and the capture-probe conjugated microspheres were resuspended in 20 μl of TE pH 8.0 and stored at 4°C in a dark box (stable for at least 6 months). The microspheres were diluted in TE buffer and counted using a Bürker chamber under the microscope at 100× magnification.

### miRNA labeling

At the beginning, 1 μg of small RNA molecules (< 200-nt) and 200 pg of a synthetic pre-labeling control RNA (5'-UCUUAGUCUUGAUUGUGGCAAUG-3', PRIMM) were mixed in order to control target preparation efficiency and to normalize expression data. The mixture was polyadenylated using Poly(A) Tailing Kit (Ambion) according to the manufactures' instructions. The reaction was precipitated with NaOAc 3 M pH 5.5 (1/10 volume) and absolute ethanol (4 volumes) overnight at -20°C. The polyadenylated RNA molecules were resuspended in 15 μl of H_2_O RNase free and then volume was reduced to 3.2 μl by vacuum (VR-1, Heto-Holten, Denmark). miRNAs labeling was performed by mRAP modified protocol in which miRNA-derived cDNAs were flanked by synthesized oligomers at each end. The SMART (switching mechanism at the 5'-end of RNA templates of reverse transcriptase) oligo sequence (SMART-16attB1-T3: 5'-TACAAAAAAGCAGGCTAATTAACCCTCACTAAAggg-3') and the overhang of the oligo-dT_15_-T7 primer (5'-GTGAATTGTTAATACGACTCACTATAGGCGC [dT]_15_N-3') were used for first strand synthesis. First strand cDNA synthesis was performed from 500 ng of small RNA in a 10 μl reaction. Then, the reaction was diluted 1:2 and incubated at 72°C for 7 min. Second strand reaction mix was added to 3.0 μl of diluted first strand cDNA to give a final concentration of 1X BD Advantage 2 PCR reaction buffer (Clontech Laboratories, Mountain View, CA, USA), 0.2 mM dNTPs, 100 nM primers (T3-biotinylated forward primer: 5' - biotAATTAACCCTCACTAAAGGG-3' and T7 reverse primer: 5'- TAATACGACTCACTATAGG-3') and 1X of Advantage 2 DNA polymerase mix (Clontech Laboratories) in a total volume of 25 μl. This second strand reaction mixture was incubated for 22 cycles of the following steps: 15 sec 95°C, 20 sec 51°C and 20 sec 72°C. Only those ss cDNAs having a SMART anchor sequence at the 5'-end were used as template and exponentially amplified. The second strand reaction was precipitated in sodium acetate-ethanol solution and dissolved in 11 μl TE buffer pH 8.0 (10 mM TrisHCl pH 8.0, 1 mM EDTA). Biotinylated cDNA quantity was assessed by Nanodrop spectrophotometer (Nanodrop Technologies) and stored at -20°C until hybridization with microspheres. Labelled target produced by a single PCR reaction was sufficient for two hybridization reactions.

### Hybridization of targets to capture probes coupled to microspheres

The microspheres of each probe set were resuspended by vortexing for approximately 20 seconds. A microsphere mixture was prepared by diluting coupled stocks to 150 microspheres of each set/μl in 1.5× TMAC Hybridization Buffer (5 M tetramethylammonium chloride, 0.15% Sarkosyl; 75 mM Tris-HCl pH 8.0; and 6 mM EDTA, pH 8.0), followed by vortex mixing for approximately 20 seconds. Two μg of biotinylated DNA target in 17 μL of TE buffer pH 8.0 was added to 33 μl of microsphere mixture (approximately 5,000 beads per color) in the wells of a 96-well plate. 17 μl of TE buffer pH 8.0 were added to background wells. Each well reaction was mixed gently by pipetting up and down several times and the labeled DNA was denatured by heating at 95-100°C for 3 min. The hybridization mixture was incubated at 48°C for 17 h, covering the plate to prevent evaporation, in a Eppendorf microplate incubator with shaking speed of 700 rpm. After incubation, the hybridization mixture was spun down for 3 min at 3,000×g to pellet the microspheres. Supernatant was carefully removed with a pipette without disturbing the microspheres. During centrifugation, fresh reporter mix was prepared by diluting streptavidin-conjugated R-phycoerythrin (Invitrogen) to 3 mg/ml in 1× TMAC Hybridization Buffer and 75 μl of reporter mix were added to the microspheres. The solution was gently mixed by pipetting and incubated in the dark at 48°C for 15 minutes in a Eppendorf microplate incubator. 50 μl of each sample were transferred to a Multiscreen HTS plate (Millipore) and analyzed on the BioPlex™ (Luminex^® ^100™, BioRad) machine at hybridization temperature.

### Bead-based detection

Each set of microspheres was distinguished by assigned colour code (different percentage of red and orange) inside the microspheres. In our experiments we have used four different probe sets (regions: 1, 21, 51 e 57). The fluorescence associated to the surface of each bead, corresponding to the amounts of bound miRNAs, was detected and measured by the laser detector. The Bio-Plex™ (Luminex^® ^100™, Bio-Rad Laboratories) system detects fluorescent dyes with an excitation wavelength of -532 nm and emission wavelength -580 nm. For each experiment, 100 events of each subset of microspheres were analyzed on the Bio-Plex™ system to obtain a median fluorescence intensity value (MFI) that was representative of the whole population of each set of beads.

### Computational analyses (data processing and quality control)

To eliminate bead-specific background, the reading of every bead for every samples was first processed by subtracting the average readings of that particular bead in the absence of target miRNAs. Samples with median fluorescence intensity values smaller than background signals were removed. Every samples was assayed in three wells. Each of the three wells contained 4 probes: miR-23a, miR-27a, miR-199a and one pre-labeling control (Spike-18). Expression data were scaled according to the pre-labeling control in order to normalize readings from different probe/bead sets for the same sample and to normalize for the labeling efficiency. Technical replicate samples for each probe were summarized by their mean profile and expression data (test/control) were log2 transformed. The error associated to each probe is obtained by quadratic propagation from standard deviation.

### qRT-PCR TaqMan

TaqMan^® ^MicroRNA Assays incorporate a target-specific stem-loop, reverse transcription primer. The stem-loop structure provides specificity only for the mature miRNA target and forms a RT primer/mature miRNA-chimera that extends the 5'-end of the miRNA. The resulting longer RT amplicon presents a template amenable to standard real-time PCR using TaqMan Assays [22]. In brief, according to the manufacture's instructions (Applied Biosystems), each 15 μl RT reaction contained purified 10 ng of total RNA, 3.0 μl of 5× stem-loop RT primer, 1× RT buffer, 0.25 mM each of dNTPs, 50 U MultiScribe™ reverse transcriptase and 3.8 U RNase inhibitor. The reactions were incubated in a Mastercycler EP gradient S (Eppendorf) in 0.2 ml PCR tubes for 30 min at 16°C, 30 min at 42°C, followed by 5 min at 85°C, and then held at 4°C. RT products were diluted two times with H_2_O prior to setting up PCR reaction. Each real-time PCR for each miRNA assay (10 μl volume) was carried out in triplicate, and each 10 μl reaction mixture included 1 μl of diluted RT product, 5 μl of 2 × TaqMan^® ^Universal PCR Master Mix and 0.5 μl of 20× TaqMan^® ^MicroRNA Assay. The reaction was incubated in a 7500 Real-Time PCR System (Applied Biosystems) in 96- well plates at 95°C for 10 min, followed by 40 cycles of the following steps: 95°C for 15 sec and 60°C for 1 min. The threshold cycle (CT) is defined as the fractional cycle number at which the fluorescence exceeds the fixed threshold of 0.2. To evaluate differences in miRNA expression, a relative quantification method was chosen where the expression of the miRNA target is standardized by a non-regulated small non-coding RNA used as reference.

Consequently, three replicates of each sample and endogenous control were amplified. U6B small nuclear (RNU6B) was used as endogenous control because the level of this small RNA remains essentially constant from sample to sample. To calculate the relative expression ratio, the 2^-Δ ΔCt ^(RQ, relative quantification) method implemented in the 7500 Real Time PCR System software [53] was used. This method determines the change in expression of a nucleic acid sequence (target) in a test sample relative to the same sequence in a calibrator sample.

## Authors' contributions

AB performed small RNA molecules isolation, capture probes coupling to microspheres, miRNA labeling method, bead-based detection with BioPlex and computational analyses (data processing and quality control). SC performed RMS cell lines culture, small RNA molecules isolation, microarray experiments and qRT-PCR validation. SC participated in conceiving the study and in development of miRNA labelling method. LT performed RMS cell lines culture and participated in total RNA extraction. AR provided RMS cell lines and revised the manuscript. GL supervised the study, participating in the design and coordination of the work, the interpretation of the results and revision of the manuscript. CDP conceived and supervised the study, participating in the design and coordination of the work, the interpretation of data and manuscript writing. All Authors read and approved the final version of the manuscript declaring that they have no potential conflicts of interests.

## References

[B1] Engels BM Hutvagner G Principles and effects of microRNA-mediated post-transcriptional gene regulation Oncogene 2006 25 6163 6169 10.1038/sj.onc.1209909 17028595

[B2] Lippman Z Martienssen R The role of RNA interference in heterochromatic silencing Nature 2004 431 364 370 10.1038/nature02875 15372044

[B3] Zhang B Wang Q Pan X MicroRNAs and their regulatory roles in animals and plants J Cell Physiol 2007 210 279 289 10.1002/jcp.20869 17096367

[B4] Harfe BD MicroRNAs in vertebrate development Curr Opin Genet Dev 2005 15 410 415 10.1016/j.gde.2005.06.012 15979303

[B5] Xi Y Shalgi R Fodstad O Pilpel Y Ju J Differentially regulated micro-RNAs and actively translated messenger RNA transcripts by tumor suppressor p53 in colon cancer Clin Cancer Res 2006 12 2014 2024 10.1158/1078-0432.CCR-05-1853 16609010

[B6] Bartel DP MicroRNAs: genomics biogenesis, mechanism, and function Cell 2004 116 281 297 10.1016/S0092-8674(04)00045-5 14744438

[B7] Stefani G Slack FJ Small non-coding RNAs in animal development Nat Rev Mol Cell Biol 2008 9 219 230 10.1038/nrm2347 18270516

[B8] Lee RC Ambros V An extensive class of small RNAs in Caenorhabditis elegans Science 2001 294 862 864 10.1126/science.1065329 11679672

[B9] Jaubert S Mereau A Antoniewski C Tagu D MicroRNAs in Drosophila: the magic wand to enter the Chamber of Secrets? Biochimie 2007 89 1211 1220 10.1016/j.biochi.2007.05.012 17629606

[B10] Landgraf P Rusu M Sheridan R Sewer A Iovino N Aravin A Pfeffer S Rice A Kamphorst AO Landthaler M Lin C Socci ND Hermida L Fulci V Chiaretti S Foà R Schliwka J Fuchs U Novosel A Müller RU Schermer B Bissels U Inman J Phan Q Chien M Weir DB Choksi R De Vita G Frezzetti D Trompeter HI A mammalian microRNA expression atlas based on small RNA library sequencing Cell 2007 129 1401 1414 10.1016/j.cell.2007.04.040 17604727 PMC2681231

[B11] Pasquinelli AE Ruvkun G Control of developmental timing by microRNAs and their targets Annu Rev Cell Dev Biol 2002 18 495 513 10.1146/annurev.cellbio.18.012502.105832 12142272

[B12] Rana TM I lluminating the silence: understanding the structure and function of small RNAs Nat Rev Mol Cell Biol 2007 8 23 36 10.1038/nrm2085 17183358

[B13] Bentwich I Avniel A Karov Y Aharonov R Gilad S Barad O Barzilai A Einat P Einav U Meiri E Sharon E Spector Y Bentwich Z Identification of hundreds of conserved and nonconserved human microRNAs Nat Genet 2005 37 766 770 10.1038/ng1590 15965474

[B14] Lewis BP Shih IH Jones-Rhoades MW Bartel DP Burge CB Prediction of mammalian microRNA targets Cell 2003 115 787 798 10.1016/S0092-8674(03)01018-3 14697198

[B15] Wang X El Naqa IM Prediction of both conserved and nonconserved microRNA targets in animals Bioinformatics 2008 24 325 332 10.1093/bioinformatics/btm595 18048393

[B16] Reinhart BJ Slack FJ Basson M Pasquinelli AE Bettinger JC Rougvie AE Horvitz HR Ruvkun G The 21-nucleotide let-7 RNA regulates developmental timing in *Caenorhabditis elegans* Nature 2000 403 901 906 10.1038/35002607 10706289

[B17] Valoczi A Hornyik C Varga N Burgyan J Kauppinen S Havelda Z Sensitive and specific detection of microRNAs by northern blot analysis using LNA-modified oligonucleotide probes Nucleic Acids Res 2004 32 e175 10.1093/nar/gnh171 15598818 PMC545470

[B18] Berezikov E Cuppen E Plasterk RH Approaches to microRNA discovery Nat Genet 2006 38 Suppl S2 7 10.1038/ng1794 16736019

[B19] Takada S Berezikov E Yamashita Y Lagos-Quintana M Kloosterman WP Enomoto M Hatanaka H Fujiwara S Watanabe H Soda M Choi YL Plasterk RH Cuppen E Mano H Mouse microRNA profiles determined with a new and sensitive cloning method Nucleic Acids Res 2006 34 e115 10.1093/nar/gkl653 16973894 PMC1635289

[B20] Mineno J Okamoto S Ando T Sato M Chono H Izu H Takayama M Asada K Mirochnitchenko O Inouye M Kato I The expression profile of microRNAs in mouse embryos Nucleic Acids Res 2006 34 1765 1771 10.1093/nar/gkl096 16582102 PMC1421506

[B21] Schmittgen TD Jiang J Liu Q Yang L A high-throughput method to monitor the expression of microRNA precursors Nucleic Acids Res 2004 32 e43 10.1093/nar/gnh040 14985473 PMC390315

[B22] Chen C Ridzon DA Broomer AJ Zhou Z Lee DH Nguyen JT Barbisin M Xu NL Mahuvakar VR Andersen MR Lao KQ Livak KJ Guegler KJ Real-time quantification of microRNAs by stem-loop RT-PCR Nucleic Acids Res 2005 33 e179 10.1093/nar/gni178 16314309 PMC1292995

[B23] Cummins JM He Y Leary RJ Pagliarini R Diaz LA Jr Sjoblom T Barad O Bentwich Z Szafranska AE Labourier E Raymond CK Roberts BS Juhl H Kinzler KW Vogelstein B Velculescu VE The colorectal microRNAome Proc Natl Acad Sci USA 2006 103 3687 3692 10.1073/pnas.0511155103 16505370 PMC1450142

[B24] Lu J Getz G Miska EA Alvarez-Saavedra E Lamb J Peck D Sweet-Cordero A Ebert BL Mak RH Ferrando AA Downing JR Jacks T Horvitz HR Golub TR MicroRNA expression profiles classify human cancers Nature 2005 435 834 838 10.1038/nature03702 15944708

[B25] Jay C Nemunaitis J Chen P Fulgham P Tong AW miRNA profiling for diagnosis and prognosis of human cancer DNA Cell Biol 2007 26 293 300 10.1089/dna.2006.0554 17504025

[B26] Yin JQ Zhao RC Morris KV Profiling microRNA expression with microarrays Trends Biotechnol 2008 26 70 76 10.1016/j.tibtech.2007.11.007 18191262

[B27] Rosa A Brivanlou AH MicroRNAs in early vertebrate development Cell Cycle 2009 in press 19875943 10.4161/cc.8.21.9847

[B28] Kloosterman WP Plasterk RH The diverse functions of microRNAs in animal development and disease Dev Cell 2006 11 441 450 10.1016/j.devcel.2006.09.009 17011485

[B29] Asli NS Pitulescu ME Kessel M MicroRNAs in organogenesis and disease Curr Mol Med 2008 8 698 710 10.2174/156652408786733739 19075669

[B30] Esquela-Kerscher A Slack FJ Oncomirs: microRNAs with a role in cancer Nature Rev Cancer 2006 6 259 269 10.1038/nrc1840 16557279

[B31] Croce CM Causes and consequences of microRNA dysregulation in cancer Nat Rev Genet 2009 10 704 714 10.1038/nrg2634 19763153 PMC3467096

[B32] Drakaki A Iliopoulos D MicroRNA Gene Networks in Oncogenesis Curr Genomics 2009 10 35 41 10.2174/138920209787581299 19721809 PMC2699834

[B33] Latronico MV Condorelli G MicroRNAs and cardiac pathology Nat Rev Cardiol 2009 6 419 29 10.1038/nrcardio.2009.56 19434076

[B34] Pauley KM Chan EK MicroRNAs and their emerging roles in immunology Ann N Y Acad Sci 2008 1143 226 239 10.1196/annals.1443.009 19076353

[B35] Bentwich I Avniel A Karov Y Aharonov R Gilad S Barad O Barzilai A Einat P Einav U Meiri E Sharon E Spector Y Bentwich Z Identification of hundreds of conserved and nonconserved human microRNAs Nat Genet 2005 37 766 770 10.1038/ng1590 15965474

[B36] Li SC Pan CY Lin WC Bioinformatic discovery of microRNA precursors from human ESTs and introns BMC Genomics 2006 7 164 10.1186/1471-2164-7-164 16813663 PMC1526439

[B37] Earley MC Vogt RF Jr Shapiro HM Mandy FF Kellar KL Bellisario R Pass KA Marti GE Stewart CC Hannon WH Report from a workshop on multianalyte microsphere assays Cytometry 2002 50 239 42 10.1002/cyto.10140 12360572

[B38] Luminex xMAP Technology http://www.luminexcorp.com/technology/index.html

[B39] Blenkiron C Goldstein LD Thorne NP Spiteri I Chin SF Dunning MJ Barbosa-Morais NL Teschendorff AE Green AR Ellis IO Tavaré S Caldas C Miska EA MicroRNA expression profiling of human breast cancer identifies new markers of tumor subtype Genome Biol 2007 8 R214 10.1186/gb-2007-8-10-r214 17922911 PMC2246288

[B40] Taylor JD Briley D Nguyen Q Long K Iannone MA Li MS Ye F Afshari A Lai E Wagner M Chen J Weiner MP Flow cytometric platform for high-throughput single nucleotide polymorphism analysis BioTechniques 2001 30 661 666 668-669 11252801 10.2144/01303dd04

[B41] Prabhakar U Eirikis E Davis HM Simultaneous quantification of proinflammatory cytokines in human plasma using the LabMAP assay Journal of immunological methods 2002 260 207 218 10.1016/S0022-1759(01)00543-9 11792390

[B42] Mano H Takada S mRAP, a sensitive method for determination of microRNA expression profiles Methods 2007 43 118 122 10.1016/j.ymeth.2007.04.006 17889798

[B43] Takada S Mano H Profiling of microRNA expression by mRAP Nature protocols 2007 2 3136 3145 10.1038/nprot.2007.457 18079713

[B44] Merlino G Helman LJ Rhabdomyosarcoma-working out the pathways Oncogene 1999 18 5340 348 10.1038/sj.onc.1203038 10498887

[B45] Shingara J Keiger K Shelton J Laosinchai-Wolf W Powers P Conrad R Brown D Labourier E An optimized isolation and labeling platform for accurate microRNA expression profiling RNA 2005 11 1461 1470 10.1261/rna.2610405 16043497 PMC1370829

[B46] Anderson J Gordon T McManus A Mapp T Gould S Kelsey A McDowell H Pinkerton R Shipley J Pritchard-Jones K Detection of the *PAX3-FKHR *fusion gene in paedriatic Rhabdomyosarcoma: a reproducible predictor of outcome? Br J Cancer 2001 85 831 35 10.1054/bjoc.2001.2008 11556833 PMC2375077

[B47] De Pittà C Tombolan L Albiero G Sartori F Romualdi C Jurman G Carli M Furlanello C Lanfranchi G Rosolen A Gene expression profiling identifies potential relevant genes in alveolar rhabdomyosarcoma pathogenesis and discriminates *PAX3-FKHR *positive and negative tumors Int J Cancer 2006 118 2772 81 10.1002/ijc.21698 16381018

[B48] Romualdi C De Pittà C Tombolan L Bortoluzzi S Sartori F Rosolen A Lanfranchi G Defining the gene expression signature of rhabdomyosarcoma by meta-analysis BMC Genomics 2006 7 287 10.1186/1471-2164-7-287 17090319 PMC1636648

[B49] Davicioni E Finckenstein FG Shahbazian V Buckley JD Triche TJ Anderson MJ Identification of a PAX-FKHR gene expression signature that defines molecular classes and determines the prognosis of alveolar rhabdomyosarcomas Cancer Res 2006 66 6936 46 10.1158/0008-5472.CAN-05-4578 16849537

[B50] Romualdi C Vitulo N Del Favero M Lanfranchi G MIDAW: a web tool for statistical analysis of microarray data Nucleic Acids Res 2005 33 Web Server W644 9 10.1093/nar/gki497 15980553 PMC1160257

[B51] Saeed AI Bhagabati NK Braisted JC Liang W Sharov V Howe EA Li J Thiagarajan M White JA Quackenbush J TM4 microarray software suite Methods in Enzymology 2006 411 134 93 10.1016/S0076-6879(06)11009-5 16939790

[B52] Tusher VG Tibshirani R Chu G Diagnosis of multiple cancer types by shrunken centroids of gene expression Proc Natl Acad Sci USA 2001 98 5116 121 10.1073/pnas.091062498 12011421 PMC124443

[B53] Livak KJ Schmittgen TD Analysis of relative gene expression data using real-time quantitative PCR and the 2(-Delta Delta C(T)) Method Methods 2001 25 402 408 10.1006/meth.2001.1262 11846609

